# Ambient AI Scribes as Emerging Infrastructure in the Learning Health System

**DOI:** 10.1002/lrh2.70115

**Published:** 2026-08-10

**Authors:** Taofeeq Oluwatosin Togunwa, Jodyn Platt

**Affiliations:** ^1^ Department of Learning Health Sciences University of Michigan Medical School Ann Arbor Michigan USA; ^2^ Michigan Institute for Data & AI in Society University of Michigan Ann Arbor Michigan USA

**Keywords:** AI governance, ambient AI scribes, clinical documentation, health system infrastructure, learning health systems

## Abstract

Ambient artificial intelligence (AI) scribes are systems that automatically generate clinical documentation from clinician‐patient conversations and are being deployed at accelerating pace across US health systems. Early evaluations report reduced documentation burden, improved clinician well‐being, and perceived efficiency gains, reinforcing a narrative of inevitability. Yet this frontline framing understates a more consequential issue: ambient scribes outsource the “first mile” of clinical documentation, thereby reshaping the production of clinical data and the learning health systems (LHSs) that depend on documentation as foundational infrastructure. This paper argues that ambient AI scribes should be understood not merely as workflow tools, but as emerging infrastructure that will materially shape the capacity and capabilities of LHSs. Drawing on infrastructure studies and LHS frameworks, we conceptualize clinical documentation as the epistemic substrate through which encounters are translated into analyzable data that power quality measurement, predictive modeling, clinical decision support, and institutional learning. When this translation is algorithmically mediated by proprietary systems, design choices, training data, and integration pathways can introduce systematic documentation errors that propagate downstream, often invisibly, through analytic pipelines. Synthesizing emerging evidence, we highlight risks including hallucinated clinical details, omission of safety‐critical information, and differential performance across patient populations with diverse accents or speech patterns. These risks mirror classic infrastructural properties described by Star: embeddedness, dependence on the installed base, wide propagation, and visibility primarily upon breakdown. From this perspective, ambient scribes may quietly reshape documentation norms, data quality, and learning trajectories well before downstream effects are routinely assessed. We conclude by outlining a governance agenda grounded in LHS principles: documentation‐quality metrics, drift monitoring, equity‐focused evaluation, transparency, and multi‐stakeholder stewardship. Without such oversight, ambient AI scribes risk stabilizing an infrastructural layer that delivers short‐term relief while eroding the long‐term integrity, equity, and trustworthiness of learning health systems.

## 1 Introduction

Ambient artificial intelligence (AI) “scribes” systems that record clinician‐patient conversations and automatically generate clinical notes, are being deployed at unprecedented speed in US health systems [1]. Tools such as Nuance DAX Copilot, Abridge, and other generative AI‐based documentation assistants have expanded rapidly across large academic centers, integrated delivery networks, and multisite physician groups [2, 3]. In one large integrated practice group more than 2.5 million patient‐encounter notes were generated within a year following deployment [4]. Early multicenter evaluations consistently show that ambient AI documentation reduces after‐hours charting, improves perceived documentation‐related well‐being, and may reduce clinician burnout at scale [5, 6]. In an environment where documentation burden is framed as an existential threat to care quality, retention, and financial stability, AI scribe adoption appears not merely attractive but inevitable.

Yet despite this intense commercial and institutional momentum, far less attention has been paid to the systemic and infrastructural implications of outsourcing the first mile of clinical documentation (the moment when the clinical encounter becomes clinical data) to proprietary AI systems. This matters because the first mile of documentation is where clinical reality is translated into institutional knowledge. Algorithmic mediation at this stage can introduce systematic biases, standardizations, or omissions that propagate across registries, dashboards, and models, effectively steering learning health systems along paths that reflect infrastructural constraints rather than clinical truth. Most published studies focus on frontline outcomes such as workflow satisfaction, burnout reduction, or self‐reported efficiency [7]. These are important but incomplete indicators of successful adoption.

What remains largely unexamined is how ambient AI scribes reshape what gets written into the electronic health record (EHR), how it is written, and by whom. These questions matter because EHR documentation is not only a legal and billing artifact; it is the epistemic substrate of learning health systems (LHSs) [8]. Errors introduced at this layer therefore carry direct patient safety implications, not only for downstream analytics but for the clinicians and patients who depend on accurate documentation to guide care. Emerging evidence raises concerns that AI‐generated notes may introduce hallucinated clinical details, omit safety‐critical information, or differentially misrepresent encounters with certain patient groups, especially individuals with diverse accents, nonstandard speech patterns, or limited English proficiency [1, 9, 10].

Structured and unstructured data from clinician notes feed population health dashboards, quality measures, risk stratification models, clinical decision support, and the next generation of AI tools [11]. The documentation layer is therefore foundational infrastructure and not simply interchangeable software. If AI‐mediated distortions enter the EHR at scale, they risk introducing new, systematic sources of documentation error that degrade the data on which learning health systems depend. The central problem, therefore, is not simply whether ambient AI scribes reduce documentation burden. The deeper issue is that they are silently becoming part of the infrastructure of clinical knowledge production, despite limited transparency, weak governance, and minimal evaluation of downstream impacts on health outcomes, equity, and cost.

This paper proceeds by first framing ambient AI scribes through the lens of health‐system infrastructure and articulating why their rapid, opaque integration poses a foundational challenge to data quality and equity. It then argues for the necessity of structured evaluation and governance of these systems, grounded in learning health system principles, stakeholder engagement, and multilevel (micro–meso–macro) analysis. The paper concludes by proposing research and policy recommendations to ensure that ambient AI scribes strengthen, rather than destabilize, the long‐term integrity of the learning health system.

## 2 Reframing the Problem: Ambient Scribes as Infrastructure

This commentary adopts a conceptual approach that synthesizes existing literature on ambient AI scribes and situates them within two bodies of theory: infrastructure studies and learning health system principles [12, 13]. This reframing shifts the focus from short‐term workflow benefits to the long‐term consequences for data quality, equity, and institutional learning.

### 2.1 Ambient Scribes as Infrastructural Components of Documentation Infrastructure

Infrastructure studies offer a well‐established lens for analyzing socio‐technical systems that become embedded in routine practice yet remain consequential for how work is organized and how knowledge travels. Star [14] conceptualize infrastructure not as a static artifact but as a relational accomplishment; they identified nine properties of infrastructure, four of which are most salient for understanding ambient AI scribes: embeddedness in practice, reliance on an installed base, propagation across interconnected systems, and visibility upon breakdown. These properties explain why infrastructural systems often produce consequential effects that go unnoticed until failure surfaces [15]. This framework emerged from foundational work in information systems and socio‐technical systems and has been repeatedly reused and extended, including in large‐scale reviews of infrastructure scholarship that show Star's criteria remain a dominant analytic foundation [16]. In health and technology research, synthesis work has explicitly operationalized Star's criteria to characterize “infrastructure‐like” phenomena (e.g., legitimacy) and to explain why effects are often observable only when tensions surface [17]. Empirically, healthcare studies using this lens highlight (i) how infrastructural properties can drive unintended transformations in care processes [18], (ii) the substantial, often under‐credited work of “infrastructuring” required to stand up federated research/data networks [19], and (iii) the inertia and constraints of installed bases that shape stepwise evolution of health information infrastructures [20].

Drawing on this framework, ambient AI scribes display fundamental infrastructural properties that are often overlooked when evaluated solely as workflow tools. Here, we use infrastructural component to emphasize that ambient scribes are a discrete socio‐technical module within the broader documentation infrastructure; however, because they mediate the “first mile” of documentation at scale, they may also solidify into an emerging infrastructural layer for clinical knowledge production These characteristics, including embeddedness and invisibility, reliance on the installed base, propagation across systems, and visibility upon breakdown, are summarized in Table 1 and illustrate how AI scribes function as infrastructural components within health systems. For example, given that AI scribes are increasingly integrated into tightly coupled clinical workflows, their outputs become invisible and simply accepted as “the note,” unless an error is egregious enough to break the illusion. This invisibility is characteristic of infrastructure: tools that become so embedded in practice that they shape work in ways that remain unnoticed until they fail.

**TABLE 1:** Infrastructural characteristics of ambient AI scribes.

Infrastructural characteristic	Definition (infrastructure theory)	Manifestation in ambient AI scribes
Embeddedness and invisibility	Infrastructure is embedded within everyday work practices and becomes invisible when functioning smoothly, surfacing mainly during breakdowns.	Ambient AI scribes operate in the background of clinical encounters, automatically generating notes that are often accepted as “the note” unless a conspicuous error (e.g., hallucinated exam findings) draws attention.
Reliance on the installed base	New infrastructure builds upon and inherits constraints from existing technical, organizational, and social systems.	AI scribes inherit EHR templates, billing logic, specialty‐specific documentation norms, and the uneven performance of speech‐recognition engines, shaping how encounters are captured and structured.
Propagation across systems	Changes in infrastructure can have cascading effects across interconnected systems and practices.	Small shifts in AI‐generated documentation (e.g., phrasing, omissions, structured fields) can propagate into quality dashboards, clinical decision support alerts, billing levels, registries, and downstream predictive models.
Visibility upon breakdown	Infrastructure becomes visible primarily when it fails, revealing otherwise hidden dependencies and risks.	Early deployments show sporadic hallucinations or omissions that clinicians detect inconsistently, suggesting a substantial volume of invisible, unrecognized documentation error during routine use.

Understanding ambient AI scribes as infrastructure helps explain why governance must extend beyond frontline usability to include data standards, drift monitoring, and equity considerations.

### 2.2 Path Dependence and Reverse Salients in Ambient AI Scribes

Building on the infrastructural properties summarized in Table 1, ambient AI scribes also inherit deeper infrastructural dynamics shaped by path dependence and reverse salients. Early adoption decisions (vendor selection, depth of EHR integration, specialty rollout) create lock‐in that influences documentation patterns and analytic pipelines [21]. Importantly, improvements in model accuracy over time do not eliminate these risks. Infrastructure theory suggests that once systems become tightly integrated into workflows, early design and integration decisions can continue to shape data production even as individual components improve [22]. As a result, technical gains may often coexist with persistent structural vulnerabilities in documentation quality, governance, and downstream learning. Prior experience with speech‐recognition systems illustrates these risks: SR‐generated notes have shown 7%–10% word‐error rates, with clinically significant errors persisting even after clinician review, and emergency‐department studies report even higher rates [23]. Modern digital‐scribe implementations still exhibit mis‐diarization and transcription errors, demonstrating how infrastructural vulnerabilities can become entrenched. These lagging components, absence of documentation‐quality metrics, limited drift monitoring, unclear audio‐governance policies, lack of labeling, function as reverse salients that impede safe system performance. As scribes move toward routinization, proactive governance, transparency, and longitudinal oversight will determine whether ambient documentation stabilizes or distorts the learning health system.

### 2.3 The Challenge for Learning Health Systems

LHS theorists emphasize that learning is only possible when supported by stable, transparent, and adaptable infrastructure; including data standards, workflows, feedback mechanisms, and governance structures [24]. Yet, current empirical studies emphasize clinician experience and short‐term efficiency, reflecting the early stage of ambient AI scribe adoption and the still‐limited evidence base [4, 25, 26]. This relative nascency presents a critical opportunity to determine which outcomes should be evaluated before these systems become routinized at scale, including not only workflow efficiency but also effects on documentation quality, equity, downstream analytics, and learning health system performance. Few studies examine how AI‐mediated documentation alters downstream analytics or predictive models, how omissions, hallucinations, or demographic disparities propagate across data pipelines, or how AI scribes interact with billing schemas, quality measurement, and safety surveillance [27, 28]. These blind spots matter because LHSs rely on documentation as the starting point of learning health system cycles of capturing data to generate knowledge that impacts practice cycle [22] (Text S1).

## 3 Implications for Health Outcomes, Quality, and Costs

Repositioning AI scribes as infrastructure clarifies how their adoption influences health‐system performance.

### 3.1 Impact on Health Outcomes

Clinical outcomes increasingly depend on data‐driven insights: predictive models, risk stratification tools, quality dashboards, and algorithm‐enabled decision support. If ambient AI scribes introduce hallucinated clinical details or omit critical information, these distortions may propagate downstream. Topaz et al. [9] showed cases where AI notes added physical‐exam details not present in the encounter or removed important safety‐relevant symptoms, raising concerns about downstream clinical decision‐making. Speech‐recognition disparities are well documented. Koenecke et al. [29] found that error rates were twice as high for Black speakers, indicating that ambient AI scribes may systematically degrade documentation accuracy for specific patient groups; a direct threat to equitable outcomes. Downstream consequences of poor input data are exemplified by the Epic Sepsis Model, which showed poor sensitivity and high false‐alarm rates at Michigan Medicine due to data drift and documentation variability [30]. Although not caused by AI scribes, this case demonstrates how upstream documentation artifacts degrade model performance.

### 3.2 Impact on Quality of Care

Documentation is central to communication, care coordination, and safety. Without intentional oversight, ambient AI scribes may normalize documentation styles that emphasize templated or generic text over individualized nuance. This concern should be understood relative to an already imperfect documentation baseline. Existing documentation practices carry well‐documented risks, including copy‐forward errors that propagate inaccurate information across encounters and standardized documentation that can reduce note specificity and clinical signal [31, 32]. The question is not whether AI‐mediated documentation is uniformly better or worse than human‐authored notes, but how it alters the distribution, scale, and visibility of documentation errors. AI‐mediated errors may be more uniform across encounters and less visually salient than clinician‐authored errors, making them harder to detect during routine review and more likely to propagate silently through downstream pipelines. In early deployments at Mass General Brigham and Emory, clinicians reported reduced documentation time but subtle concerns that notes could become overly structured or formulaic, potentially missing clinically relevant “soft data” such as affect, uncertainty, or nuanced patient narratives [33]. Studies of auto‐generated templated notes (pre‐generative AI) show increased note bloat and decreased signal‐to‐noise ratio [34]. Generative AI scribes may reduce bloat but can still lose contextual information if summaries compress inaccurately. In mental‐health settings, automated transcription often misrepresents emotional tone, altering interpretation or continuity [35].

### 3.3 Impact on Costs

Cost impacts extend beyond subscription fees to downstream financial risks associated with documentation errors, billing variability, regulatory exposure, and turnover linked to clinician burnout. AI‐mediated documentation may increase revenue in the short term by improving capture of billable elements or supporting higher‐complexity coding. However, such gains must be weighed against downstream financial risks, including increased audit exposure, compliance vulnerability, and the potential misalignment between coded documentation and clinical reality when documentation becomes algorithmically optimized. At scale, these dynamics may shift costs rather than eliminate them, particularly in systems that rely on documentation‐derived data for quality measurement and risk adjustment. Large evaluations (e.g., Abridge pilots at Sutter Health and Emory) found significant reductions in “pajama time,” suggesting savings from reduced burnout and improved retention; critical when clinician turnover may cost systems $500 000–$1 million per physician [3]. However, documentation inaccuracies can alter billing levels or generate compliance vulnerabilities. Poor documentation inputs also degrade predictive models, producing costly alert fatigue, unnecessary testing, or missed deterioration, thus, illustrating how upstream documentation errors generate real downstream costs.

## 4 Tensions Across Micro, Meso, and Macro Levels

Ambient AI scribes generate multi‐level tensions requiring different governance mechanisms. Table 2 maps these tensions across micro (clinicians, patients), meso (health systems), and macro (vendors, regulators) levels and proposes governance pathways.

**TABLE 2:** Sociotechnical tensions and governance gateways for ambient AI scribes in learning health systems.

Level/concept	Core tension or issue	Analytical description (underlying dynamics, risks, and implications)	Governance gateways/mitigation strategies
Micro‐level (clinicians and patients)	Clinician relief vs. clinician overreliance	Ambient AI scribes reduce burnout and enhance face‐to‐face engagement, yet overreliance on AI‐generated text may weaken situational awareness, reduce reflective practice, and shift clinicians toward passive acceptance of documentation.	Reversible integration pathways; specialty‐specific accuracy reviews; clinician‐facing error dashboards; maintaining hybrid documentation options.
	Patient trust vs. limited consent transparency	Continuous ambient audio capture creates a mismatch between patient expectations and technical realities regarding storage, processing, and model retraining. This produces significant trust tensions and potential consent invalidation.	Explicit patient‐consent gateways; transparent explanations of audio retention and model use; community advisory oversight.
Meso‐level (Health Systems, Departments, Quality & Safety Units)	Workflow efficiency vs. data‐quality degradation	Health systems adopt AI scribes to relieve burden, but weak oversight may introduce documentation discrepancies that propagate through registries, safety analytics, and CDS pipelines.	AI Documentation Stewardship Committees; routine error auditing; integration of scribe metadata into quality systems; drift monitoring.
	Rapid deployment vs. limited governance capacity	Pressures to rapidly reduce burnout outpace organizational capacity for post‐deployment surveillance, bias monitoring, or model performance auditing.	Investment in internal informatics capacity; structured rollout protocols; staged approval processes (“scribe formularies”).
Macro‐level (Vendors, Regulators, Policy Environment)	Vendor opacity vs. transparency needs	Proprietary constraints limit access to training data, demographic performance, and hallucination rates, conflicting with LHS transparency requirements.	Model‐agnostic labeling standards; required telemetry reporting; contractual transparency obligations.
	Innovation speed vs. regulatory lag	Generative documentation tools lack clear FDA classification, validation norms, or labeling requirements, leaving governance gaps.	Regulatory frameworks for post‐market monitoring; reporting standards for AI documentation tools.

## 5 Stakeholders, Policy Priorities, and the Path Forward

As the documentation layer becomes algorithmically mediated without oversight, the LHS may evolve on a distorted data foundation, without any systematic mechanism for monitoring learning. The result is an infrastructure that provides short‐term labor relief but undermines data quality, equity, and trust in the long term. Such hidden infrastructural drift becomes a threat to the integrity of the entire learning enterprise.

Governing ambient AI scribes as emerging infrastructure requires coordinated engagement across clinicians, patients, health‐system leaders, vendors, and regulators. Clinicians experience both the benefits and risks of these tools, yet early evaluations also document hallucinated or omitted details that can silently enter the EHR [9]. Patients likewise face equity and trust concerns: long‐standing evidence of higher speech‐recognition error rates for Black speakers underscores the need for community involvement in consent, transparency, and monitoring. At the system level, leaders must balance pressure for rapid deployment with limited governance capacity, while predictive‐model failures linked to distributional mismatch between training and deployment data (e.g., the Epic Sepsis Model at Michigan Medicine) highlight downstream vulnerabilities that vendors and regulators must address through transparency and standards.

A forward‐looking agenda should prioritize several interconnected goals. Developing metrics for scribe‐mediated data quality will require defining what accurate documentation looks like in practice, likely through structured clinician review against source audio, patient‐reported accuracy under open notes frameworks, and comparison against established documentation standards such as those used in medical audits. Longitudinal studies of demographic disparities, mixed‐methods research on micro–meso–macro dynamics, and patient‐centered consent models are equally essential. Patients represent an underutilized resource for quality assessment, particularly as open notes policies give patients direct access to their clinical documentation and a legitimate stake in its accuracy. Importantly, policy frameworks must address the retention, governance, and permissible use of source audio recordings from clinical encounters, balancing quality assurance needs against patient privacy. Ultimately, safe adoption depends on multi‐stakeholder stewardship that aligns ambient AI scribes with the learning health system's core commitments to data fidelity, equity, and continuous improvement.

## Author Contributions

T.O.T. conceived the study, conducted the conceptual analysis, and drafted the manuscript. J.P. provided senior supervision, contributed to the conceptual framing and interpretation, and critically revised the manuscript for important intellectual content. Both authors approved the final version of the manuscript.

## Funding

The authors have nothing to report.

## Conflicts of Interest

The authors declare no conflicts of interest.

## Supporting information


**Text S1:** Definition of learning health systems and the learning health cycle.

## Data Availability

Data sharing is not applicable to this article, as no datasets were generated or analyzed during the current study.
