# Supplementary material for: Ambient AI Scribes as Emerging Infrastructure in the Learning Health System
Source: Learn Health Syst. 2026 Aug 10;10(4):e70115. doi: 10.1002/lrh2.70115 (PMC13456971; doi:10.1002/lrh2.70115)
Supplement: Supplementary file 1 [file LRH2-10-e70115-s001.docx]

**Supplemental Material**

**Supplementary Text 1: *Definition of Learning Health Systems and the Learning Health Cycle***

Learning health systems (LHSs) are health systems designed to enable continuous improvement by systematically transforming data generated through routine clinical care into knowledge that informs practice, policy, and system redesign. In an LHS, clinical operations and research are tightly coupled: data from everyday care are captured, analyzed, and translated into actionable insights, which are then reintegrated into clinical workflows, creating iterative feedback loops between practice and learning.

The learning health cycle is commonly conceptualized as a progression from data → knowledge → practice, followed by reassessment and refinement. Patient encounters generate structured and unstructured data through clinical documentation, diagnostic testing, and administrative processes. These data are aggregated and analyzed using statistical methods, quality improvement frameworks, and increasingly, machine learning and artificial intelligence tools. The resulting knowledge (such as performance metrics, predictive models, or clinical guidelines) is then used to inform decision-making, clinical workflows, and policy interventions. Subsequent clinical activity generates new data, allowing the cycle to repeat and enabling continuous system-level learning.

Crucially, the effectiveness of an LHS depends on the fidelity, completeness, and equity of the underlying data inputs. Clinical documentation plays a foundational role in this process, as it serves as the primary mechanism through which patient encounters are rendered legible to health systems and downstream analytic infrastructure. Distortions introduced at this initial stage; whether through omissions, inaccuracies, or systematic biases; can propagate throughout the learning cycle, shaping analytics, decision support, and institutional learning in ways that may be difficult to detect or correct once embedded.
